# Arginase and Arginine Decarboxylase – Where Do the Putative Gate Keepers of Polyamine Synthesis Reside in Rat Brain?

**DOI:** 10.1371/journal.pone.0066735

**Published:** 2013-06-19

**Authors:** Daniela Peters, Jana Berger, Kristina Langnaese, Christian Derst, Vince I. Madai, Michael Krauss, Klaus-Dieter Fischer, Rüdiger W. Veh, Gregor Laube

**Affiliations:** 1 Institut für Integrative Neuroanatomie, Charité - Universitätsmedizin Berlin, Berlin, Germany; 2 Institut für Biochemie und Zellbiology, Medizinische Fakultät, Otto-von-Guericke-Universität, Magdeburg, Germany; Aston University, United Kingdom

## Abstract

Polyamines are important regulators of basal cellular functions but also subserve highly specific tasks in the mammalian brain. With this respect, polyamines and the synthesizing and degrading enzymes are clearly differentially distributed in neurons versus glial cells and also in different brain areas. The synthesis of the diamine putrescine may be driven via two different pathways. In the “classical” pathway urea and carbon dioxide are removed from arginine by arginase and ornithine decarboxylase. The alternative pathway, first removing carbon dioxide by arginine decarboxlyase and then urea by agmatinase, may serve the same purpose. Furthermore, the intermediate product of the alternative pathway, agmatine, is an endogenous ligand for imidazoline receptors and may serve as a neurotransmitter. In order to evaluate and compare the expression patterns of the two gate keeper enzymes arginase and arginine decarboxylase, we generated polyclonal, monospecific antibodies against arginase-1 and arginine decarboxylase. Using these tools, we immunocytochemically screened the rat brain and compared the expression patterns of both enzymes in several brain areas on the regional, cellular and subcellular level. In contrast to other enzymes of the polyamine pathway, arginine decarboxylase and arginase are both constitutively and widely expressed in rat brain neurons. In cerebral cortex and hippocampus, principal neurons and putative interneurons were clearly labeled for both enzymes. Labeling, however, was strikingly different in these neurons with respect to the subcellular localization of the enzymes. While with antibodies against arginine decarboxylase the immunosignal was distributed throughout the cytoplasm, arginase-like immunoreactivity was preferentially localized to Golgi stacks. Given the apparent congruence of arginase and arginine decarboxylase distribution with respect to certain cell populations, it seems likely that the synthesis of agmatine rather than putrescine may be the main purpose of the alternative pathway of polyamine synthesis, while the classical pathway supplies putrescine and spermidine/spermine in these neurons.

## Introduction

Polyamines (putrescine, spermidine and spermine) at physiological pH-values are positively charged molecules and interact with nucleic acids and proteins. Consequently, they are involved in a large variety of biological functions, often linked with cell growth, survival, and proliferation. Most interesting, they even contribute to aging and longevity [1], [2]. In addition, in the brain they serve a variety of tissue specific roles influencing neuronal excitability by modulating ion channels and receptors [3], [4], [5], [6], [7]. They contribute to the complex rectification of Kir channels in retinal Müller cells and enhance propagation of molecules within the glial syncytium [8], [9]. Even under pathological conditions like stroke [10], [11] epilepsy [12], [13], or mental disorders [14], [15], the polyamine system is highly responsive.

Given a non-homogeneous distribution of polyamines [16], [17] as well as polyamine pathway enzymes [18], [19], [20], [21] in the brain, it seems likely that physiological and pathological actions of polyamines will at least partially depend on regional rather than systemic effects. The polyamines spermidine/spermine were localized to astrocytes and neurons [17]. However, since polyamine pathway enzymes like ornithine decarboxylase and spermidine synthase are predominantly expressed in neurons, astrocytes most likely serve as stores, clearing the extracellular space from excess polyamines. This regulatory role is strongly supported by data showing an efficient uptake of haptenylated spermine by rat brain astrocytes in acute slices (R.W. Veh et al, unpublished).

The cellular redistribution of polyamines and their highly regulated synthesis and degradation render the localization of polyamine pathway enzymes as an rational approach for revealing the involvement of the polyamine system in local circuits like the cerebellar cortex [21]. The synthesis of polyamines in distinct cell types may involve two different pathways via ornithine and agmatine, respectively, both leading to the formation of the diamine putrescine. Since agmatine is seemingly involved with neurotransmission [22], [23], [24] it is currently not known whether the agmatine pathway is additionally used to fuel putrescine and hence spermidine/spermine synthesis. With this regard, the comparative analysis of arginase (Arg; EC 3.5.3.1) and arginine decarboxylase (ADC; EC 4.1.1.19) expression, the enzymes responsible for ornithine and agmatine synthesis, respectively, may help to appraise the potential of individual cell types for utilizing either one or both pathways. Assuming that spermidine/spermine and not putrescine are more important for brain-specific polyamine functions, the comparison with spermidine synthase (SpdS; EC 2.5.1.16) expression on the one hand and agmatinase (Agm; EC 3.5.3.11) expression on the other hand can be expected to provide insight into local mechanisms involving spermidine/spermine and/or agmatine.

We therefore raised and characterized polyclonal antibodies against arginase and arginine decarboxylase and used the affinity-purified antibodies to localize these enzymes in the rat brain. Using two other previously characterized antibodies against downstream enzymes of both pathways, spermidine synthase [20] and agmatinase [18], we analyzed and compared the respective labeling patterns in distinct rat brain areas.

## Results

The classical and the alternative pathway for putrescine synthesis are driven by two sets of enzymes, both belonging to the same two protein families, namely the arginase family (Arg, Agm) and the Orn/Lys/Arg decarboxylase class-II family (ODC, ADC). While the two families are structurally unrelated, within a family the individual members share varying degrees of sequence homology. On the amino acid level, rat Arg and Agm only show 14.6% identity in a 452 amino acid overlap. Thus, a cross reactivity of anti-Arg antibodies with Agm was not to be expected. The two arginase isoforms Arg1 and Arg2, however, share 59.4% identical amino acids in a 313 amino acid overlap. Given the relatively high overall degree of similarity between Arg1 and Arg2 isoforms, for immunization, a 211 amino acid C-terminal Arg1-sequence was chosen for immunization, sharing 65% identity with Arg2 in a 200 amino acid overlap but only 18.5% identity with Agm in a 227 amino acid overlap. Within the Orn/Lys/Arg decarboxylase class-II family, ODC and ADC are also similar, displaying 50.2% identity in a 520 amino acid overlap (LALIGN program; [32]). Therefore the C-terminal part of the ADC sequence was chosen for immunization (C-terminal 67 amino acids), showing only 25.4% identity with the C-terminus of rat ODC.

### Characterization of Anti-ADC Antibody

**Figure 1:**
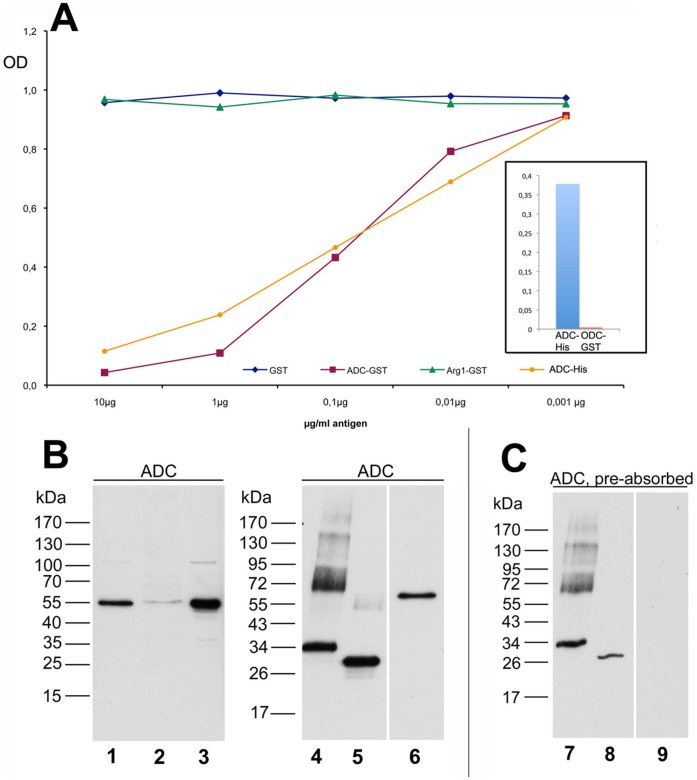
Characterization of the anti-ADC antibody. A ELISA assay displaying anti-ADC activity. In a competitive ELISA assay preincubation with ADC-GST and ADC-His fusion proteins at increasing concentrations competitively inhibited the specific interaction, demonstrating the reactivity of the antibody against ADC fusion protein. In contrast, pre-adsorption using the unrelated fusion protein (Arg1-GST) or GST did not show any effect on immunoreactivity. The inset shows a direct ELISA, illustrating the activity of the anti-ADC antibody against the immunogen ADC as compared to a potentially cross-reacting ODC partial fusion protein. At the same dilution as used for the competitive assay (1∶30.000), no cross-reactivity was observed. B-C Characterization of the anti-ADC antibody by Western blotting. B: Strong protein bands at 55 kDa were detected in rat liver (lane 1 and 6) as well as in rat prostate tissue (lane 3). In rat brain (lane 2), at the same molecular weight a faint but distinct band was observed. Moreover, the antibody strongly reacted with the bacterially expressed partial fusion proteins ADC-GST (36,7 kDa, lane 4) as well as ADC-His (30,7 kDa, lane 5). C: Pre-absorption of the antibody with 10 µg/ml ADC-His purified fusion protein clearly attenuated the intensity of the fusion protein bands (lane 7, 8) and led to a complete disappearance of the ADC signal in rat liver homogenate (lane 9), thus demonstrating the specificity of the antibody. Loading: Lane 1, rat liver homogenate (25 µg); lane 2, rat total brain homogenate (50 µg); lane 3, rat prostate homogenate (25 µg); lane 4 and 7, ADC-GST fusion protein (25 ng); lane 5 and 8, ADC-His fusion protein; lane 6 and 9, rat liver homogenate (25 µg).

In a competitive ELISA assay (Fig. 1A), with increasing concentrations of either the protein used for immunization (ADC-GST) or the corresponding 6HisTR-construct (ADC-His), the activity against ADC-His was progressively inhibited, thus demonstrating the antibody’s high reactivity against ADC. By contrast, pre-incubation with an unrelated GST-construct (Arg1-GST) and GST alone did not affect the specific activity of the affinity-purified anti-ADC antibody. Moreover, using a direct ELISA assay (Fig. 1A, inset), no cross-reactivity of the anti-ADC antibody with a C-terminal (70 amino acids) ODC-GST fusion protein was detected, neither at the dilution (1∶30,000) used for the competitive ELISA assay against His-tagged and GST fusion proteins as shown in Fig. 1A, nor at the dilution used for immunocytochemistry (1∶5,000, not shown). Thus, the above mentioned C-terminal ADC-GST fusion protein used for immunization led to the generation of an ADC antibody not cross-reacting with ODC.

**Figure 2:**
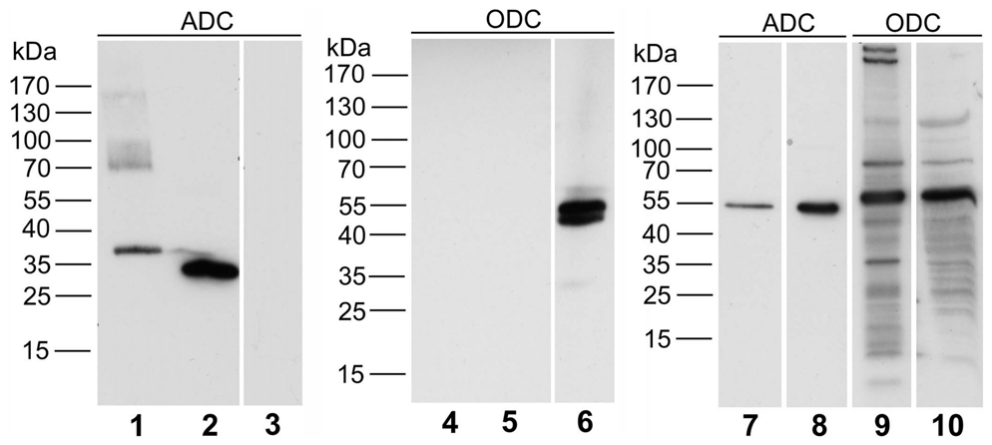
The anti-ADC antibody does not cross-react with ODC. The partial fusion proteins ADC-GST (lane 1) as well as ADC-His (lane 2) are clearly recognized by the antibody raised against ADC. However, the antibody does not cross-react with ODC-transfected 293T cell lysate (lane 3). For control, ODC-transfected 293T cell lysate was analyzed in parallel with a commercial ODC antibody (Santa Cruz, sc-33539), resulting in a strong Western signal at the respective molecular weight (lane 6). Moreover, the ADC partial fusion proteins are not recognized by the ODC antibody (lanes 4, 5). In rat liver and prostate homogenates (lanes 7–10), protein bands of slightly different molecular weights, corresponding to the calculated molecular weights of 49 kDa (ADC) and 51 kDa (ODC), respectively, were either detected by ADC or ODC antibodies, again verifying the specificity of the ADC antibody for ADC protein. Loading: lane 1 and 4, ADC-GST fusion protein (10 ng); lane 2 and 5, ADC-His fusion protein (10 ng); lane 3 and 6, ODC (h) 293T lysate (5 µg, Santa Cruz); lane 7 and 9, rat liver homogenate (25 µg); lane 8 and 10, rat prostate homogenate (25 µg).

The specificity of the affinity-purified anti-ADC antibody was further characterized by Western blotting (Fig. 1B, C). With tissue homogenates (Fig. 1B), the antibody displayed very strong bands in liver and prostate. By contrast, in rat brain homogenate a single distinct but faint immunoreactive band was observed at the same molecular weight, indicating a comparatively weaker expression when compared with gland tissues. For controls, the antibody was tested with bacterially expressed fusion proteins (Fig. 1B) and clearly recognized the relevant GST and His-tagged bacterial fusion proteins at the calculated molecular weights of ADC-GST (36.7 kDa), and ADC-His (30.7 kDa). The appearance of the described band observed in liver homogenate was completely abolished by pre-incubating the antibody with 10 µg/ml ADC-His purified fusion protein (Fig. 1C), thus demonstrating the specificity of the observed immunosignal. With blotted bacterial fusion proteins, immunoreactivity was clearly attenuated though not completely eliminated by antigen pre-incubation (Fig. 1C). Given the relatively high degree of similarity of ADC and ODC, we further tested a potential cross reactivity of the ADC antibody also by Western blotting (Fig. 2). However, when using ODC-transfected cell lysate no signal was obtained, whereas strong immunoreactive bands were observed using the highly immunoreactive bacterial fusion proteins ADC-GST and ADC-His as positive controls. Vice versa, using a commercial ODC antibody (Santa Cruz, sc-33539) resulted in a strong signal with ODC-transfected cell lysate while ADC bacterial fusion proteins were not recognized. For further verification of the signals observed for ADC and ODC by Western blotting, we then compared the molecular weights detected by both antibodies when using tissue homogenates from rat liver and prostate. Here, with both tissues, the subtle difference in molecular weight between ADC (49.4 kDa) and ODC (51 kDa) was unambiguously demonstrated.

### Characterization of Anti-Arg1 Antibodies

**Figure 3:**
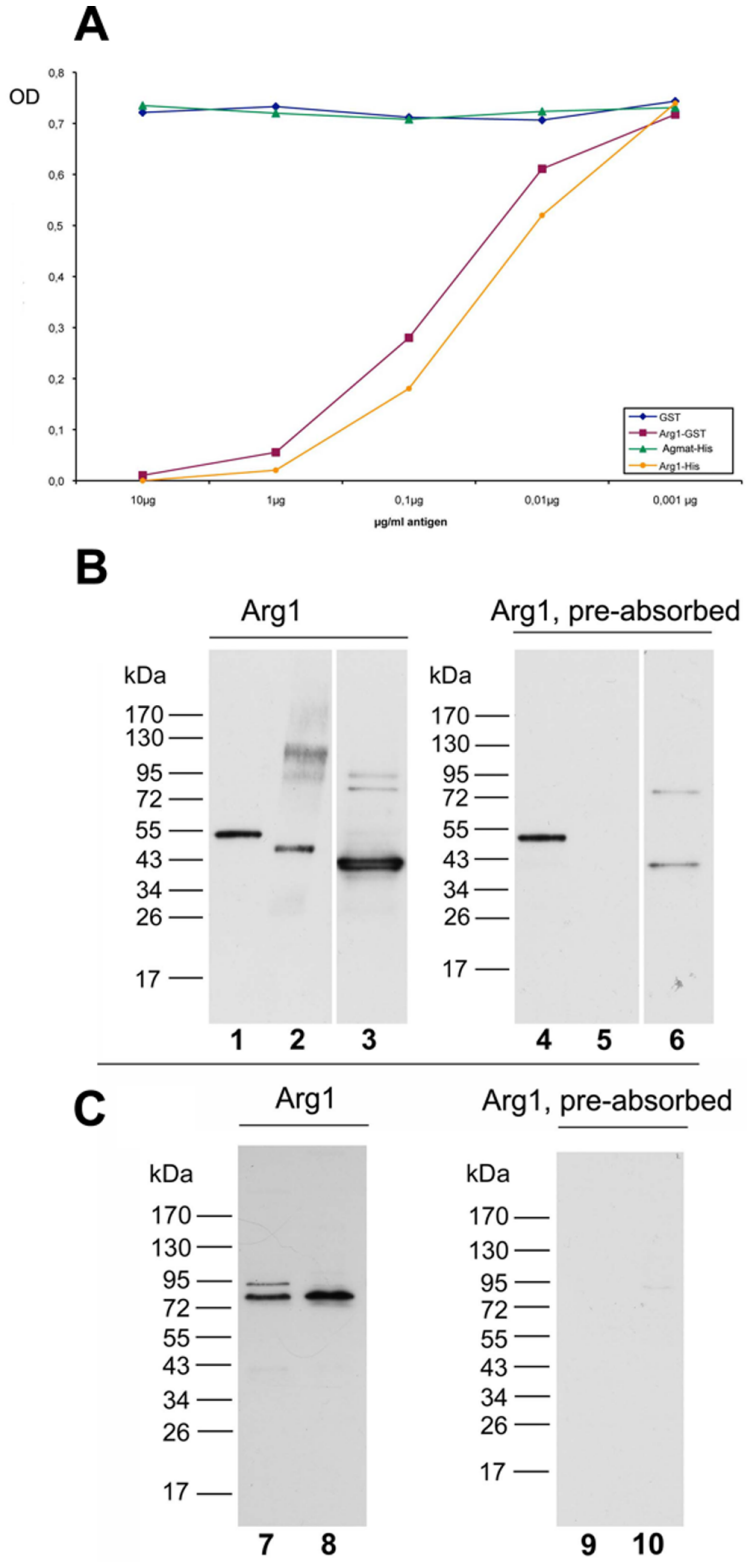
Characterization of the anti-Arg1 antibody. A ELISA assay displaying anti-Arg1 activity Competitive ELISA assay demonstrating the ability of different protein constructs to interfere with the anti-Arg1 immunosignal. Arg1-GST and Arg1-His at increasing concentrations competitively inhibited the specific interaction, demonstrating the antibodies reactivity against Arg1 fusion protein. In contrast, pre-adsorption using either Agm-His or -GST constructs did not show any effect on reactivity. B–C Characterization of the anti-Arg1 antibody by Western blotting. B: The anti-Arg1 antibody detected the bacterially expressed partial fusion proteins Arg1-GST (lane 1, 49,2 kDa) as well as Arg1-His (lane 2, 43,2 kDa). A strong 37kDa band was detectable in rat liver homogenate, a well known source rich of Arg1 (lane 3). C: In rat cortex homogenate (lane 7) and cytosol (lane 8) a band of about 80 kDa was detected. Pre-adsorption of the antibody with Arg1-His purified fusion protein (10 µg/ml lane 4–6, 40 µg/ml lane 9–10) lead to the disappearance of the observed bands, except for lane 4, which was loaded with Arg1-GST fusion protein. This reactivity could be attributed to residual anti-GST activity by testing the antibody against unconjugated GST protein (not shown). Loading: lane 1 and 4, Arg1-GST fusion protein (25 ng); lane 2 and 5, Arg1-His fusion protein (25 ng); lane 3 and 6, rat liver homogenate (25 µg); lane 7 and 9, rat cerebral cortex homogenate (40 µg); lane 8 and 10, rat cerebral cortex cytosol (40 µg).

In the competitive ELISA assay (Fig. 3A), the fusion protein constructs Arg1-GST and Arg1-His competitively inhibited the activity of the affinity purified anti-Arg1 antibody against Arg1-His with a half-maximal concentration below 0.1 µg/ml, thus demonstrating the antibody’s specific reactivity against Arg1 protein. By contrast, GST protein and the similar construct Agm-His did not affect the activity.

With Western blotting (Fig. 3B) anti-Arg1 antibody detected the respective fusion proteins Arg1-GST (49.2 kDa), and Arg1-His (43.2 kDa). In rat liver, a prominent band at 37 kDa and additional minor bands at roughly the double molecular weight were evident. By pre-adsorbing the antibody with Arg1-His protein, the observed bands were effectively abolished except for the Arg1-GST construct, due to residual anti-GST activity in the purified antibody. In brain (Fig. 3C), however, the anti-Arg antibody produced a prominent broad band at roughly the doubled calculated molecular weight at 80 kDa. The specificity of this band was verified by pre-adsorption of the antibody using Arg1-His, thus indicating the existence of a covalently linked Arg dimer in brain in contrast to liver.

### Immunocytochemical Analysis

**Figure 4:**
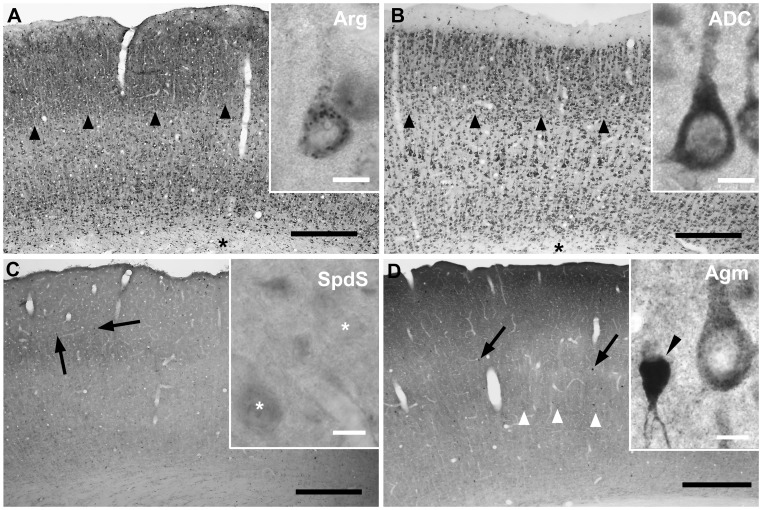
Cerebral Cortex. In the cerebral cortex, both, Arg-like and ADC-like immunoreactivity were predominantly localized to neuronal cell bodies and main dendrites of all neuronal cell types (A, B), but were also detected in the neuropil. Here, labelling intensity varied with respect to different cortical layers. With both antibodies, cortical layers II/III were comparatively most prominently labelled (arrowheads in A, B). In contrast to neurons, immunoreactivity in glial cells was less pronounced, but still clearly evident in white matter (asterisk) oligodendrocytes (also compare Fig. 3 H). Within neuronal cell bodies, Arg-like immunoreactivity mostly displayed a punctate labelling pattern suggesting a localization to subcellular compartments roughly measuring 0,5 µm in diameter (inset in A). By contrast, ADC-like immunoreactivity was strongly but diffusely distributed throughout the cytoplasm (inset in B). For comparison, the downstream enzymes of the classical and alternative pathway for polyamine synthesis, SpdS (C) and Agm (D) clearly differed with respect to neuronal labelling, since here subpopulations of interneurons were most strongly labelled (arrows in C, D; black arrowhead in inset in D). By contrast, with anti-SpdS-antibodies, principal neurons were not clearly delineated from the surrounding neuropil. With anti-Agm antibodies, however, principal neurons like pyramidal cells showed an intermediate level of labelling intensity when compared to interneurons and neuropil (inset in D; white arrowheads in D). Scale bars represent 400 µm in A–D, 10 µm in insets in A–D.

With immunocytochemistry on rat brain sections, we then screened different brain regions for the expression of anti-Arg-like and anti-ADC-like immunoreactivity and compared the resulting labeling patterns with those obtained with previously characterized antibodies against the polyamine pathway enzymes spermidine synthase [20] and agmatinase [18]. Generally, both antibodies raised against the two arginine-metabolizing enzymes broadly labeled neurons and neuropil throughout the brain, though with varying intensity and displaying different intracellular patterns of reactivity. In the cerebral cortex (Fig. 4), labeling occurred in neurons throughout all cortical layers. With anti-Arg antibodies, in the majority of neurons the cytosolic immunosignal displayed a distinct punctate pattern clearly emphasizing cell bodies rather than individual processes (Fig. 4A). By contrast, anti-ADC antibodies produced a diffuse cytosolic labeling, even more prominent in neurons when compared to the surrounding neuropil than with anti-Arg antibodies. A differential neuropil labelling, however, was also evident, especially when increasing the antibody concentration above values necessary to display neuronal cell bodies (see below). In cerebral cortical layers II/III, the neuropil immunoreactivity was more pronouced than in the underlying layers IV–VI. By comparison, SpdS-like and Agm-like immunoreactivities (Fig. 4C, D) were clearly different with respect to an intense labeling of distinct populations of interneurons in contrast to only low to moderate levels of labeling in principal neurons like pyramidal cells.

**Figure 5:**
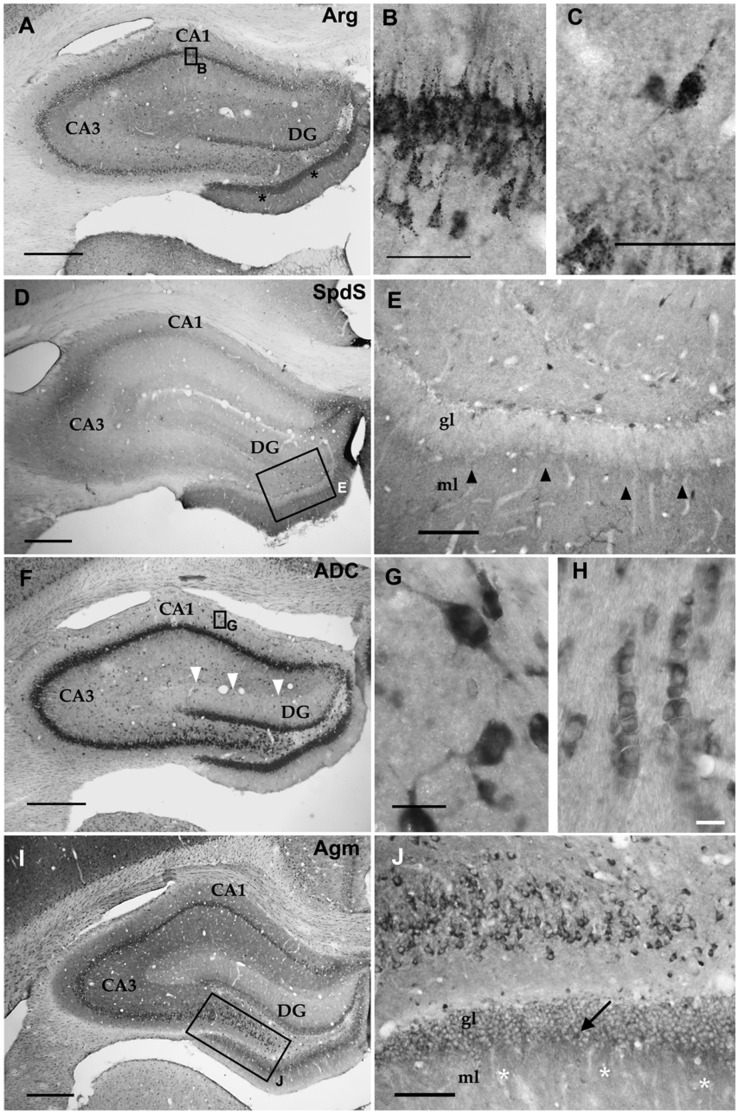
Hippocampus. In the hippocampal formation, similarly to the cerebral cortex, immunoreactivity for Arg (A, B, C) and ADC (F, G, H) was generally present in principal neurons (A, B, F), many interneurons (A, C, F, G), and stacks of white matter oligodendrocytes (H). Similarly, the neuropil was also differentially labelled in the dentate gyrus (DG, asterisks in A) and ammons horn (CA, white arrowheads in F). With this respect, Spds and Agm also displayed different labelling intensities (D, I black arrowheads in I and white asterisks in J). Cell body labelling for SpdS and Agm was most pronounced in interneurons (compare Fig. 2 C, D). With anti-Arg labelling most principal neurons and interneurons displayed the typical cytosolic punctate labelling pattern, in contrast to ADC labelling. The boxed areas in A, D, F, I are shown at higher magnifications in B, E, G, J, respectively. ml = molecular layer, gl = granule cell layer. Scale bars represent 400 µm in A, D, F, I; 100 µm in E; 50 µm in C,D; 20 µm in G; 10 µm in H.

In the hippocampal formation (Fig. 5), Arg and ADC labelling clearly displayed the ammons horn (CA) pyramidal cell layers and dentate gyrus (DG) granule cell layer (Fig. 5A, F). Besides principal neurons, numerous interneurons were also immunoreactive (Fig. 5B, C, G). Similar to the cerebral cortex, SpdS and Agm immunoreactivities were more prominent in interneurons and neuropil areas (Fig. 5D, E, I, J) than in principal neurons. In addition, ADC and also Agm were also clearly expressed in oligodendrocytes (Fig. 5F, H, I). With anti-Arg labeling, in addition to the majority of immunoreactive neurons displaying the before mentioned punctate intracellular pattern, also some strongly but diffusely labeled hippocampal interneurons were observed.

**Figure 6:**
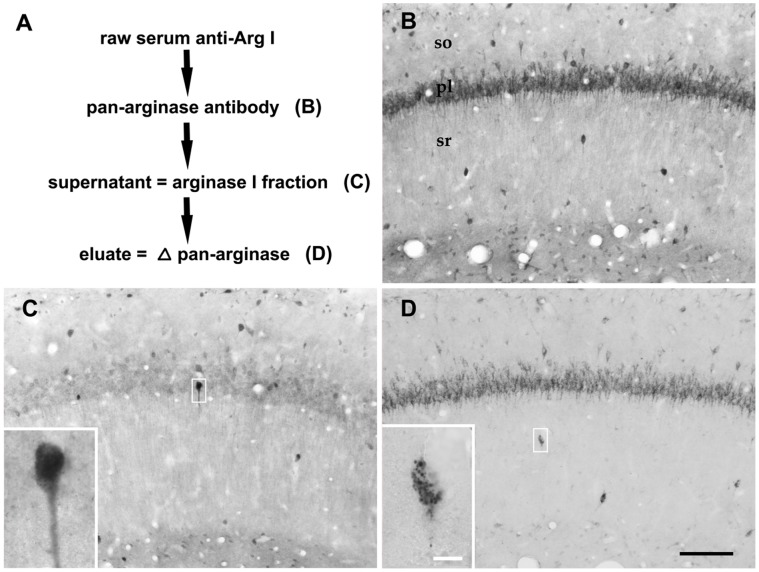
Pre-absorption of affinity-purified anti-Arg1-antibodies with Arg2- fusion protein (C-terminal 223 amino acids). To verify the potential cross reactivity between the two isoforms Arg1 and Arg2, the anti-Arg1 antibody was incubated with Arg2-recombinant protein-loaded nitrocellulose membranes and subsequently eluated (A). The resulting fractions, supernatant (C) and eluate (D), were compared with the original affinity-purified reagent (presumed “pan-arginase”-antibody; B). In the hippocampal CA1 region, two populations of immunoreactive cells became evident. The Arg II membrane-eluated fraction labelled the majority of neuronal cell bodies in both, principal neurons and interneurons (D) with interneurons located mostly outside the pyramidal cell layer. In contrast, the presumed Arg I-representing supernatant predominantly displayed a separate population of putative interneurons (C), several of them among CA1 pyramidal cells. In contrast to the typical punctate labelling pattern as observed with the eluate (inset in D), the scattered interneurons displayed in the inset in (C) were strongly but diffusely labelled. so = stratum oriens; pl = pyramidal cell layer; sr = stratum radiatum. Scale bars represent 100 µm in B–D; 10 µm in insets.

Given the relatively high degree of amino acid identity between the two isoforms Arg1 and Arg2, we aimed to identify whether the different intracellular labelling could reflect a cross reactivity of the antibody, raised against the C-terminus of Arg1, with Arg2. For this purpose, we subjected the affinity purified anti-Arg1 antibody to pre-incubation with the membrane-bound, potentially cross-reacting C-terminus of Arg2. Assuming that the not cross-reactive fraction of the antibody remaining in the supernatant would preferentially display Arg1, we tested supernatant and eluate on hippocampal sections (Fig. 6). Indeed, the population of interneurons with a diffusely labeled cytoplasm was largely separated (Fig. 6C) from the majority of typically “spotted” neurons (Fig. 6C, D), the latter reflecting pan-arginase (presumably Arg2-preferring) labelling (Fig. 6D).

**Figure 7:**
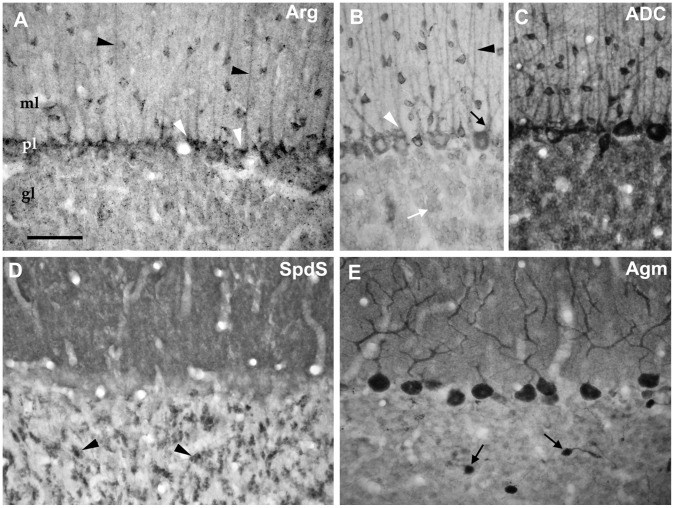
Cerebellar cortex. A: Arg-like immunoreactivity was observed in neurons and neuropil in all cerebellar cortical layers but was most obvious in cell bodies (white arrowheads) and processes (black arrowheads) of Bergmann glial cells. GABAergic interneurons including basket, stellate, and Golgi cells were also observed displaying a prominent cytosolic punctate labelling pattern, whereas Purkinje and granule cells displayed considerably less label. B, C: By contrast, ADC-like immunoreactivity was most prominent in cell bodies and branched dendritic trees (black arrow) of Purkinje cells and inhibitory interneurons in the molecular and granule cell layer. However, Bergmann glial cell bodies (white arrowhead) and processes (black arrowhead) and granule cells (white arrow) also were positive. With increasing antibody concentrations (compare 1∶5000 in B and 1∶1000 in C), the diffuse immunoreactivity in the neuropil became clearly evident. D: With SpdS labelling, cell bodies were hardly delineated. Instead, the neuropil of the molecular layer was strongly but diffusely immunopositive, while in the granule layer numerous giant mossy fiber-like boutons (arrowheads) were detected. E: Agm labelling was also markedly expressed in the molecular layer but most prominently observed in Purkinje cell bodies and denritic processes. Comparatively strong labelling was evident in the regionally occuring unipolar brush cells (arrows). ml = molecular layer, pl = Purkinje cell layer, gl = granule cell layer. Scale bar represents 50 µm.

Within the cerebellar cortex (Fig. 7), the cellular sources for agmatine and spermidine synthesis differed strikingly with respect to the expression in principal versus interneuron populations. With respect to the “classical” pathway enzymes Arg and SpdS, Purkinje cells were not particularly noticeable (Fig. 7A, D), while ADC and especially Agm prominently displayed the cell bodies and dendritic trees of these neurons. Both, Arg and ADC, were also detected in Bergman glial cells (Fig. 7A, B, C). In the molecular layer of the cerebellar cortex, the labelling pattern suggested a strong expression of the enzymes of both pathways in synaptic compartments of this layer. Giant mossy fibre boutons [21] were, however, only clearly delineated with anti-SpdS antibodies.

**Figure 8:**
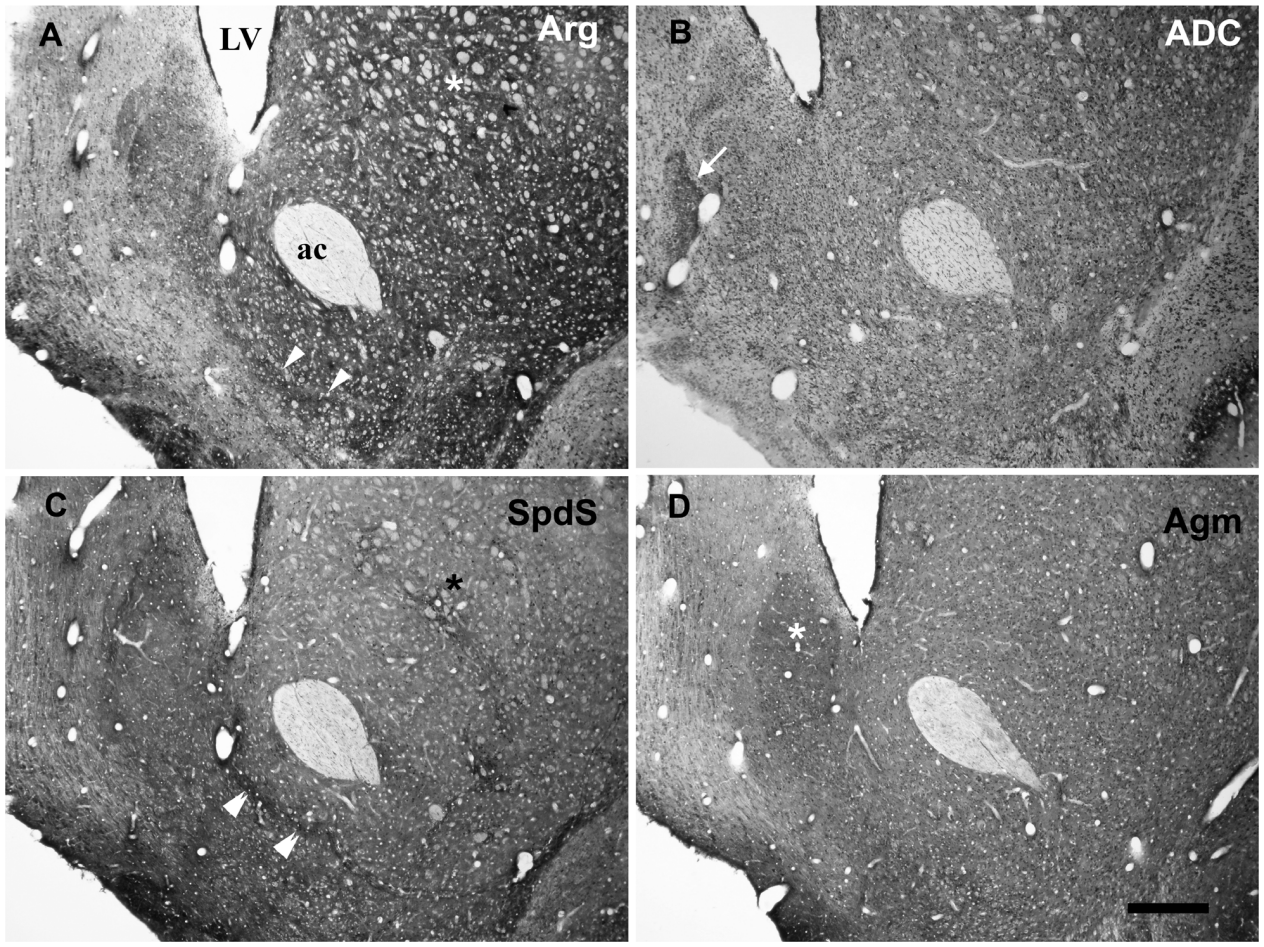
Basal forebrain at the level of the anterior commissure. In this area containing the nucleus accumbens, the “classical” and “alternative” pathway enzymes Arg/SpdS (A, C) and ADC/Agm (B, D), respectively, clearly differed with respect to the homogeneity of labelling in the neuropil. With anti-Arg and anti-SpdS antibodies, patches of varying labelling intensity became evident in the Acb (arrowheads in A; double arrowheads in C) and striatum (asterisks in A, C). By contrast, ADC- and Agm-like immunoreactivity was comparatively homogeneous in these neuropil regions, most obviously displaying neuronal cell bodies. However, in the dorsal accumbens shell area, Agm labelling was pronounced in the neuropil (asterisk in D) and surrounded by stronger staining for Arg- and SpdS-like immunoreactivities. Note the prominent ADC-like immunoreactivity in the neighbouring major island of Calleja (arrow in B) resulting from the high density of labelled cell bodies in this area. LV = lateral ventricle ac = anterior commissure. Scale bar represents 400 µm.

Since antibodies against SpdS are known to demarcate patches of strongly labelled neuropil in basal ganglia areas like the striatum and nucleus accumbens [20], we compared the overall labelling patterns in the basal forebrain (Fig. 8). While in this area the agmatine pathway enzymes were relatively uniformly distributed with respect to neurons and neuropil, except for ADC in the septal and Agm in the dorsal accumbens shell region (Fig. 8B, D), Arg and SpdS both displayed a more differentiated neuropil in striatal and ventromedial accumbens shell regions (Fig. 8A, C).

**Figure 9:**
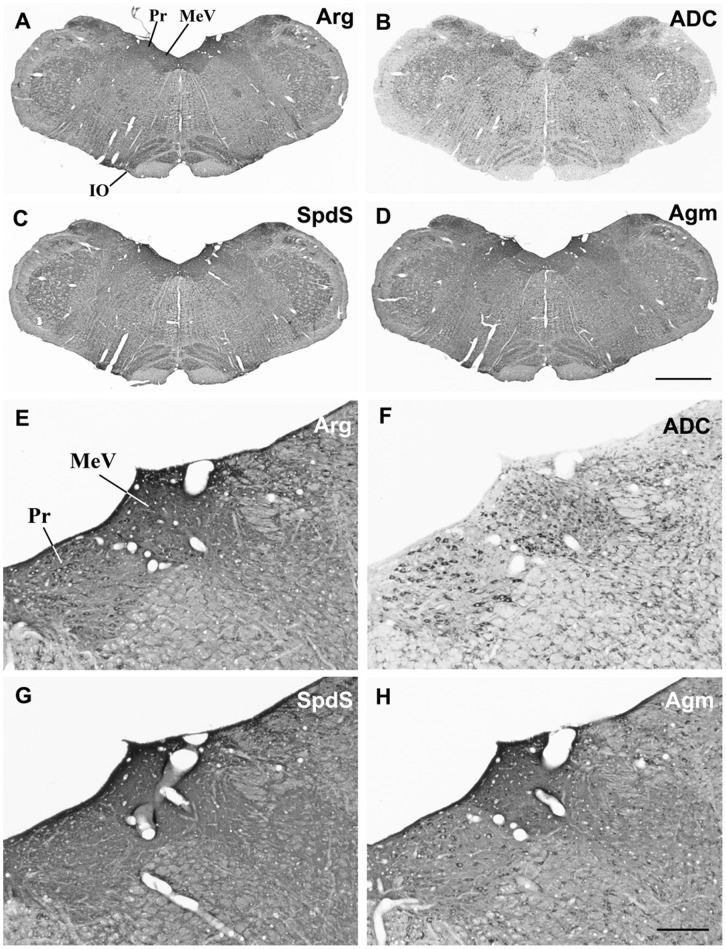
Brain stem. In the brain stem, the overall labelling patterns were very similar for all tested antibodies. In survey micrographs (A–D), several brain stem nuclei like the medial vestibular nucleus (MeV), the nucleus prepositus hypoglossi (Pr), and the inferior olive (IO) appear clearly pronounced. In addition to neuropil labelling in these areas, with anti- Arg and anti-ADC antibodies, numerous neuronal cell bodies were also evident (E, F) in the MeV. By contrast, in the Pr, cell body labelling was evident with all antibodies, though less pronounced with anti-SpdS antibodies. Scale bars represent 1000 µm in A–D; 200 µm in E–H.

In the brainstem, a robust Agm expression was previously reported in the medial vestibular nucleus [18], a relay between vestibular receptors and eye muscles or spinal cord [33] and the nucleus prepositus hypoglossi involved with the coordination of eye movements. At this brain stem level, the immunocytochemical distribution patterns for the enzymes from both pathways showed a remarkable degree of similarity (Fig. 9 A to 9 D). However, SpdS did not obviously highlight neuronal cell bodies when compared to the three other enzymes, which were also prominently expressed in giant neurons of the reticular formation and in the inferior olive.

**Figure 10:**
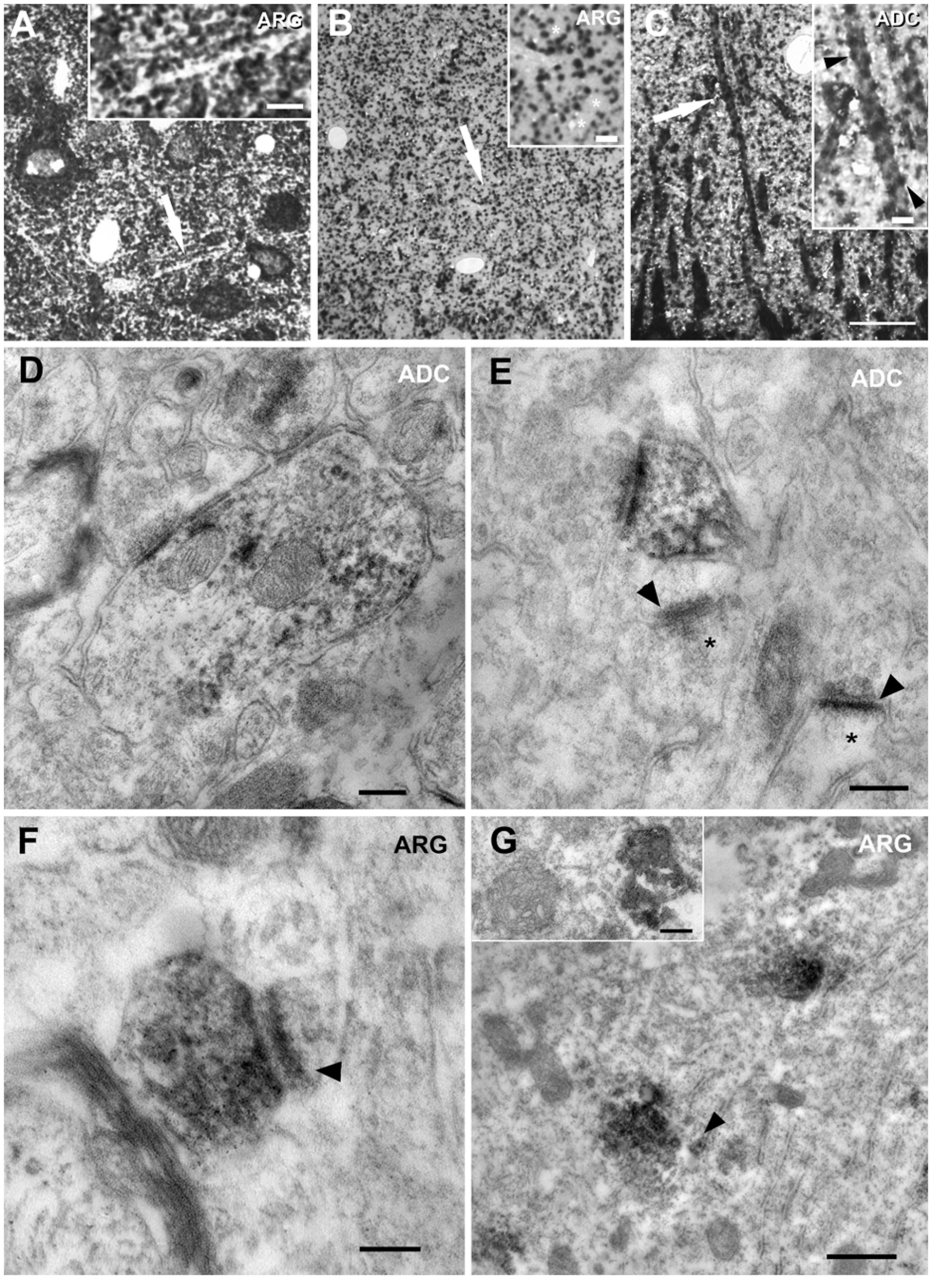
Virtual pre-embedding and electron microscopy. To obtain more information about the abundant diffuse neuropil labelling as observed with standard immunoperoxidase/DAB light microscopy, here we used the high sensitivity VirP labelling for light microscopy (A–C) and DAB-based immune-electron microscopy (D–G). With VirP, a clearly structured neuropil displaying numerous punctate profiles became evident in cerebral cortex (A) and hippocampus (B, C). The areas labelled by arrows are displayed at higher magnification in the insets in A–C. The labelled punctate profiles partly delineated dendritic profiles (asterisk in inset in B; arrowheads in inset in C), thus suggesting a synaptic localization of the antigens. With electron microscopy, ADC was localized to dendrites (D) and dendritic spines (E; compare unlabelled spines marked by asterisks), while Arg-like immunoreactivity was detected in presynaptic spine terminals (F). Postsynaptic densities of asymmetrical spine synapses are indicated by arrowheads. In neuronal cell bodies (G), the frequently displayed Arg-like punctate immunoreactivity was localized to numerous Golgi compartments including pre-Golgi buds at the endoplasmic reticulum (arrowhead in G). Rarely, immunoreactivity could also be attributed to ruptured mitochondria. Scale bars represent 20 µm in A–C; 5 µm in insets in A–C; 200 nm in D, E, inset in G; 100 nm in F; 500 nm in G.

Previous experiments analyzing the fine structural localization of Agm in rat cerebral cortex neuropil [29] demonstrated that the diffuse signal observed with standard immunoperoxidase light microscopy correlates with a punctate pattern of immunoreactivity obtained when virtual pre-embedding (VirP) involving a CARD signal amplification was used. We therefore postulated that the neuropil labelling observed here with antibodies against Arg and ADC may similarly be displayed at higher resolution using the VirP procedure with light microscopy. Indeed, in the cerebral cortex (Fig. 10A) and hippocampus (Fig. 10B, C), numerous punctate profiles were observed. Within the neuropil, some of these punctae were clearly associated with dendritic profiles, suggesting either a presynaptic (terminal) or postsynaptic (dendritic spine) localization of the antigen. Besides this neuropil labeling, both antibodies also labeled neuronal cell bodies as observed with standard DAB light microscopy.

With electron microscopy visualizing DAB-based immunoperoxidase reactivity, ADC was localized to dendrites (Fig. 10D) and dendritic spines (Fig. 10E), whereas Arg was detected in presynaptic terminals (Fig. 10F) in a fraction of cortical synapses. Within neuronal cell bodies, ADC-like immunoreactivity was associated with ER-like and vesicular structures (not shown), while Arg was distinctly observed in Golgi compartments (Fig. 10G). Rarely, also labeled disrupted mitochondria were visualized with anti-Arg antibodies (Fig. 10G, inset).

## Discussion

From theoretical reasoning there are two nominal pathways leading to the formation of putrescine, the starting point in spermidine/spermine synthesis: the “classical” pathway via ornithine and the “alternative” pathway via the breakdown of the putative neurotransmitter agmatine. The relevance of both pathways, however, has been challenged, primarily based on data concerning the two arginine-converting enzymes Arg and ADC [34], [35]. Arg, existing in two isoforms (Arg1, Arg2), apparently is not necessary to maintain polyamine homeostasis in Arg1/Arg2 single and double knockout mice [35]. However, Arg1 knockout animals die at the age of two weeks due to hyperammonemia resulting from an impaired urea cycle [36]. Dietary spermidine/spermine and also agmatine were discussed to account for the maintenance of blood and tissue polyamine levels [35], [37].

In the brain the situation may be different due to the blood-brain barrier. Apparently, spermidine/spermine only have a very limited capacity to cross the blood-brain barrier [38]. By contrast, agmatine is readily transported into the cerebrospinal fluid [39]. Furthermore, intravenously administered arginine rather takes effect by acting as a cerebrospinal fluid than blood precursor of agmatine [39]. Thus, the brain seems to be able to locally synthesize the putative neurotransmitter agmatine. Hence, the systemic effects observed with impaired arginine metabolism may obscure any brain specific effects.

With respect to Arg1 and Arg2 isoforms, our data indicate that both Arg isoforms are expressed in the brain. Furthermore, two groups of immunocytochemically differentiated hippocampal interneurons were clearly different with respect to the cytosolic distribution of the reaction product. The assumed Arg1-specific fraction of our anti-Arg1 antibody diffusely labelled the cytoplasm in a subset of interneurons. Thus, this assumption is supported by the confirmed cytosolic expression of Arg1, the prevalent isoform in the liver, in contrast to mitochondrial localization of Arg2. A limited expression of Arg1 in the brain may be beyond the detection limits of the initial Northern blot experiments, demonstrating Arg1 in liver only, in contrast to a widespread distribution of Arg2, including the brain [40], [41]. Similarly, in a study on mouse arginase isoforms [42] with Northern blots Arg1 was detected clearly only in liver and very faintly in brain, while Arg2 was observed in kidney but not in the brain. With in situ hybridization, however, Arg2 was broadly detected in rat [43]and mouse [42] brains. In contrast to earlier studies, Arg1 was more strongly expressed in the mouse brain than Arg2.

In the rat brain in situ hybridization data [43] demonstrating hippocampal and cerebellar Arg2 expression are in good agreement with our immunocytochemical results, especially with respect to the lack of detectable expression in cerebellar Purkinje cells. Immunocytochemically, Arg1 was abundantly reported in the mouse brain, however these experiments were performed using a commercial antibody, which according to the manufacturers information, was not sufficiently characterized for immunocytochemical applications. This antibody had been raised against a human Arg1 sequence, sharing 61,5% identity in a 156 amino acid overlap with rat Arg2 [42]. Unfortunately, there are no published data available reassessing the immunocytochemical localization in the brain using this antibody after the Arg1 knockout mouse became available in the same laboratory [44]. Since the breeding of Arg1 and Arg2 knockout mice apparently was discontinued, we also were not able to test our antibodies against tissues of such animals.

Using the highly sensitive VirP method, Arg immunoreactivity was also widely but distinctly displayed in neuropil compartments away from the soma. Here, the observed presynaptic localization argues in favour of a synaptic role for polyamine synthesis. Interestingly, SpdS previously was also observed in presynaptic terminals [21]. As mentioned above, Arg2 is regarded as a mitochondrial enzyme, exhibiting a N-terminal targeting sequence [45]. With our antibody, however, we only rarely detected a mitochondrial localization. This might be due to a limited penetration of the antibodies across mitochondrial membranes during pre-embedding labelling. In our hands the somatic immunosignal was preferentially associated with the Golgi apparatus including pre- and post-Golgi vesicles.

Similar to Arg-like immunoreactivity, ADC-like immunoreactivity was also observed distant from the soma, though at postsynaptic rather than presynaptic sites. The proposed role for mouse ADC as an antizyme inhibitor [46] would not necessarily be expected to work in synaptic compartments. Furthermore, given the broad expression of inactive monomeric ODC and regulatory antizyme I [47], [48], e.g. in cerebellar Purkinje cells, it seems unlikely that additionally an antizyme inhibitor would also be broadly constitutively expressed in the brain.

Agmatine in the brain may be derived from dietary rather than from internal sources, such as plants and intestinal microorganisms [37]. However, among various foodstuffs analyzed by HPLC, agmatine was mostly not detectable and only present in soybeans at relatively high concentrations [49]. Assuming an important role for agmatine as a neurotransmitter, the local expression of a synthesizing enzyme such as ADC as well as a degrading enzyme like Agm [29], in synaptic compartments may not only seem reasonable but may also be necessary to subserve brain specific functions.

Considering the potential role for ADC in fuelling an alternative pathway for polyamine synthesis, the localization patterns for Arg/SpdS and ADC/Agm as observed here argue against this assumption and many central neurons apparently express the enzymes for both pathways. In contrast, agmatine is an important neurotransmitter and its regulation seems to be involved with mood disorders [15], [50], [51]. Thus, regulating agmatine levels may be the intrinsic purpose of the alternative pathway, with agmatinase rather being an agmatine inactivator instead of a putrescine supply. Deciphering the precise physiological relevance of polyamines in brain circuits in health and disease may in future shed more light on the currently still highly speculative discussion about the various roles of these organic polycations in the central nervous system.

## Materials and Methods

### Molecular Biology

In order to produce arginine decarboxylase (ADC) fusion proteins we PCR-amplified a C-terminal fragment of rat ADC cDNA. XhoI-tagged primers (XhoI-5′-gacacaaagtcccttctcgg-3′, XhoI-5′-gaagggacaggatttatgttgc-3′) were developed according to GeneBank Acc. No. NM_001014261, spanning nt 1325 to nt 1690. Our PCR-product, however, differed from the above mentioned rat sequence by containing additional 46bp, leading to an altered reading frame and therefore altered C-terminal amino acid sequence. Since our sequence showed strong homology to mouse ADC (Acc. No. NM_172875), we additionally amplified and sequenced the complete open reading frame and submitted a new GeneBank entry with Acc. No. HQ413774. Accordingly, the amplified sequence used for cloning the fusion protein constructs encodes the C-terminal 67 amino acids of rat ADC (HQ413774).

For arginase-1 (Arg1) we amplified nucleotides 363–1328 of GenBank Acc. No. NM_017134 encoding the C-terminal 211 amino acids using the XhoI-tagged primers XhoI-5′-agggtccaccctgacctatg-3′ and XhoI-5′-tctccagatgcctttgcttc-3′. For arginase-2 (Arg2) we amplified the homologous region with nucleotides 497–1367 of GenBank Acc. No. NM_019168 encoding the C-terminal 223 amino acids using the XhoI-tagged primers XhoI-5′-cgacaccacccagatctctg-3′ and XhoI-5′-tcagaaacaaaaacccaacaga-3′. For control purposes a partial ornithine decarboxylase (ODC) fusion protein encoding the C-terminal 70 amino acids was cloned. For this purpose, nucleotides 1333–1652 of GenBank Acc. No. J04791 were amplified using the XhoI-tagged primers XhoI-5′-gctgctgcttctactttcaa-3′ and XhoI-5′-cctactcttacaaagacatt-3′.

The resulting PCR fragments were cloned into the bacterial expression vectors pGEX-4T-1 (Pharmacia, glutathione-S-transferase (GST) fusion protein) and pET32b(+) (Novagen, 6His-tagged thioredoxin (6His-TR) fusion protein) using *XhoI* restriction sites. Sequence and orientation of the inserted cDNA fragments of both vectors were verified by DNA sequencing. Agmatinase fusion proteins were produced as described earlier [18]. ADC-, Arg1-, Arg2-, ODC-GST-fusion proteins as well as ADC-, Arg1-, Arg2- and Agm-His- fusion proteins were over-expressed in the *E. coli* strain BL21DE3 and purified using either Glutathion-Sepharose 4B (GST, Pharmacia Biotech) or Ni^2+^-NTA agarose (TR, Qiagen) as described by the manufacturer. Table 1 summarizes the fusion proteins used for antibody production, purification and characterization.

**Table 1:** Bacterial fusion proteins used for antibody generation, purification and characterization.

Abbreviation	Protein
Agm-GST	GST fusion protein containing amino acids 176–353 from Agm sequence
Agm-His	6His-tagged thioredoxin fusion protein containing amino acids 176–353 from Agm sequence
Arg1-GST	GST fusion protein containing amino acids 113–323 from Arg1 sequence
Arg1-His	6His-tagged thioredoxin fusion protein containing amino acids 113–323 from Arg1 sequence
Arg2-GST	GST fusion protein containing amino acids 132–354 from Arg2 sequence
Arg2-His	6His-tagged thioredoxin fusion protein containing amino acids 132–354 from Arg2 sequence
ADC-GST	GST fusion protein containing amino acids 392–459 from rat ADC sequence
ADC-His	6His-tagged thioredoxin fusion protein containing amino acids 392–459 from rat ADC sequence
ODC-GST	GST fusion protein containing amino acids 393–462 from rat ODC sequence

### Antibodies

Generation of monospecific affinity-purified antibodies against Arg1 and ADC. The generation, purification, and characterization of monospecific antibodies against Arg1 and ADC was performed similarly as previously described [20]. Briefly, rabbits were immunized with purified Arg1-GST and ADC-GST, respectively, fusion protein. The activity of the crude sera was tested against the fusion proteins Arg1-His, Arg1-GST, Agm-His, ADC-His, ADC-GST and GST. The serum dilution optimal for affinity purification was determined by ELISA (Arg1 1:8,000, ADC 1∶30,000). Purification was performed in a three-step procedure. Nitrocellulose membranes were loaded with the cognate antigen Agm-His [18] for removal of potential cross-reactivities for 2 h at room temperature (RT) followed by affinity absorption on membranes loaded with either Arg1-His or ADC-His fusion protein (10 µg/ml in PBS). Generally, the different fusion protein loaded membranes were rinsed 3 times in phosphate buffered saline (PBS) and blocked with 5% normal goat serum (NGS) for 1 h at RT after loading proteins. For affinity purification, the membranes were incubated with the antiserum diluted in 5% NGS in PBS over night at 4°C. After washing, bound antibodies were eluted with 200 mM glycine-HCl, pH 2.5, supplemented with 0.9% NaCl and 0.1% bovine serum albumin (BSA) for 30 min at RT. Eluted antibodies were subsequently dialyzed against PBS and 20 mM phosphate buffer (PB), pH 6.0, at 4°C. For concentration antibodies were loaded on a SP-Sepharose fast flow column at 4°C. Antibodies were eluted in a small volume with 200 mM carbonate buffer, pH 9.0, pooled and again dialyzed against PBS. The concentrated affinity-purified antibody was aliquoted and stored frozen. Activity was determined with indirect and competitive ELISA assays [25].

### Preparation of Rat Tissue

All animal experiments were conducted in accordance with the guidelines of the European Communities Council directive 86/609/EEC and were approved by the Regional Berlin Animals Ethics Committee (LaGeSo No. G 0168/01).

For immunocytochemistry, adult male Wistar rats were deeply anaesthesized using a mixture of Ketavet (Parke-Davis) and Domitor (Pfizer). The animals were then perfused transcardially with 0.9% NaCl solution for 1 minute followed by a fixative composed of 4% paraformaldehyde, 0.05% glutaraldehyde, and 0.2% picric acid for 20 minutes. For immunofluorescence, 4% paraformaldehyde only was used as fixative. Brains were removed from the scull. Tissues were rinsed extensively in 0.1 M phosphate buffer, and freeze-protected with 1 M sucrose in 0.1 M phosphate buffer. Tissue was frozen at −60°C in hexane and stored frozen at –80°C until use. For immunocytochemistry, a total of 29 rats and 419 frontal sections were analyzed.

For Western blotting, adult male wistar rats were anaesthetized with isoflurane and killed by cervical dislocation. Liver and prostate were rapidly dissected, cut into pieces on a cold metal plate at 0°C, transferred to ice-cold homogenization buffer containing protease inhibitors (20 mM Tris, 150 mM NaCl, 1% Triton-X-100, 0,1% SDS pH 7,6, protease inhibitor cocktail tablet (Roche) and homogenized with a motor driven pistill. After incubation for 30 min on ice the homogenates were cleared by centrifugation (15 min, 20000×g, 4°C). Rat brain tissue was homogenized in buffer containing 320 mM sucrose, 5 mM HEPES pH 7.4 and protease inhibitor cocktail tablet. The homogenate was first centrifuged at 900×g for 10 min. To prepare a cytosolic fraction the supernatant was again spun at 100000×g (1 h, 4°C). Protein concentration was determined by a bicinchoninic acid assay. Specific labelling for Arg (a-Arg 1∶10000) and ADC (a-ADC 1∶10000) was reproduced 3 times and was blocked by pre-incubation with 10 or 40 µg/ml Arg1-His (1 h at RT) or 10 µg/ml ADC-His, respectively.

### Immunocytochemistry

#### Immunoperoxidas

Immunocytochemical labeling was performed according to standard diamino benzidine/nickel (DAB/nickel) immunoperoxidase protocols [26], [27], [28]. Free-floating sections were treated with 1% sodium-borohydride in PBS for 15 min, washed with PBS and incubated in a solution containing 10% NGS, 0.3% Triton X-100, and 0.05% phenylhydrazine in PBS for 30 min. Primary antibody was diluted in 10% NGS, 0.3% Triton X-100 supplemented with 0.1% sodium azide and 0.01% thimerosal and incubated for 36 h at 8°C. After washing for 1 h in PBS and another hour in PBS containing 0.2% bovine serum albumin (PBS-BSA), the sections were incubated with biotinylated secondary goat anti-rabbit antibody for 24 h at 8°C. The sections were again washed as described above and further incubated for 6 h with an avidin-biotinyl-peroxidase-complex (Vector Elite ABC kit) in PBS-BSA. After the final washing bound peroxidase was visualized in a solution containing 1.4 mM DAB, 10 mM imidazole, 6.6 mM nickel ammonium sulfate, and 0.15% H_2_O_2_ in 50 mM Tris/HCl buffer, pH 7.4. All sections were developed for 15 min. Labeled sections were mounted, dehydrated and cover-slipped with Entellan.

#### Immunocytochemical controls

For negative controls, either primary or secondary antibodies were omitted. No staining was detected under these conditions. To further verify the specificity of anti-ADC and anti-Arg-labeling, purified antibodies were preincubated with the corresponding antigen at concentrations previously determined in a competitive ELISA assay (10 or 20 µg/ml). However, already at 10 µg/ml immunocytochemical labelling of brain sections was completely blocked.

### Immunoblotting

Extracts from tissues were reduced and denatured with Roti-Load1 (Roth) by heating to 98°C for 5 min, then subjected to SDS-PAGE on 5–20% gradient gels, transferred to nitrocellulose and blocked 5% non-fat dry milk in TBST. Blots were incubated with the antibody overnight at 4°C. Unbound antibody was removed by repeated washing with TBST. The blots were then treated with peroxidase-conjugated anti-rabbit antibody (Jackson Immuno Research) at a 1∶10,000 dilution for 4 h at room temperature (RT), respectively. The blots were developed with the ECL detection system (Amersham Biosciences) according to the manufacturer’s instructions.

### Electron Microscopy

Following immunoperoxidase visualization, sections were transferred to 0.1 M PB, postfixed for 30 min with 1% osmium tetroxide in 0.1 M PB, and washed several times in PB. Finally, sections were dehydrated in a graded series of ethanol for 10 min each including block staining with 2% uranyl acetate (Serva, Heidelberg, Germany) in 70% ethanol, and flat embedded in Araldite CY212 (Serva). Thin silver sections were contrasted with uranyl acetate and lead citrate and analyzed with a LEO 912 electron microscope equipped with a slow scan digital camera (Proscan 1K).

### Virtual Pre-embedding

Virtual Pre-embedding (VirP) was performed as described in detail previously [29]. Briefly, freshly perfused brain tissue was sectioned at a thickness of 50 µm using a vibratome. Sections were rinsed in PBS, then incubated twice for 10 min in 20% sucrose diluted in 0.1 M phosphate buffer. Subsequently, the sections were transferred on a plastic support and freeze-thawed using liquid nitrogen [30]. After washing in PBS, sections were incubated in 0.1% sodium borohydride in PBS for 15 minutes. After washing in PBS, sections were pre-incubated for 30 minutes in a solution of 10% normal goat serum, 0.05% Triton-X-100 for permeabilization and 0.05% phenylhydrazine in PBS. Sections were then incubated for at least 36 hours at 4°C in the primary antibody solution (Rabbit-anti-ADC 1∶5,000; Rabbit-anti-Arginase 1∶2,000.), containing 10% NGS in PBS, 0.05% triton, 0.1% sodium azide and 0.01% thiomersal. Sections were washed in PBS, followed by pre-incubation with PBS-Albumin (PBS-A, 2% w/v bovine serum albumine in PBS) for 1 hour and incubation over night at 4°C with the biotinylated secondary antibody (biotin-goat-anti-rabbit, diluted 1∶2.000 in PBS-A). After washing and pre-incubation in PBS-A for 1 hour, sections were incubated over night at 4°C with ELITE ABC (Vector, 1∶200) in PBS-A. After sinsing, sections were pre-incubated for 15 minutes with 10 nM of tetramethylrhodamine-tyramide (TMR-T) diluted in CARD-solution (50 nM Tris-Buffer, 10 nM imidazole). TRM-T was deposited by adding hydrogen peroxide (0.0015% final concentration) for 15 minutes. For preparation of TMR-T see [29], [31]. Sections were then post-fixed with 1% osmium tetroxide in 0.1 M phosphate buffer for 10 minutes and washed in phosphate buffer. Subsequently, they were dehydrated in a graded series of ethanol including a block staining with 2% uranyl acetate in 70% ethanol for 30 minutes. Sections were incubated in 100% propylene oxide twice, followed by incubation in a 1∶1 mixture of propylene oxide and araldite for 10 min and pure araldite for 20 min. Sections were then flat-embedded in freshly prepared resin. and polymerized for 24 h at 60°C.

Semithin (500 nm) sections were cut using a diamond knife (Diatome, Switzerland) and an ultramicrotome (Reichert Ultracut S, Leica, Germany) and dried at 70°C to aminosilane-coated slides.

Semithin sections were incubated in methanolate etching solution (1 M sodium methoxide in a 2∶1 mixture of methanol and toluene) for 10 minutes, followed by rinsing in a 1∶1 mixture of methanol and toluene for 5 minutes and twice in acetone for 5 minutes. After rinsing with distilled water, slides were transferred to 100 mM acetate buffer (pH 5). Afterwards, sections were incubated in 2% hydrogen peroxide for 5 minutes, transferred again into acetate buffer and finally rinsed. Slides were then transferred into a humid chamber. The following steps were performed at room temperature. Sections were pre incubated in 10% NGS for 30 minutes, followed by incubation with rabbit anti-tetramethylrhodamine (RaTMR) diluted 1∶7.500 in 10% NGS over night. After rinsing, sections were pre-incubated in PBS-A for 15 minutes and then incubated with biotin-goat-anti-rabbit diluted 1∶1.000 in PBS-A for 4 hours. After washing, sections were incubated with ELITE ABC diluted 1∶200 in PBS-A for 1 hour. After rinsing, sections were pre-incubated in DAB-solution for 15 minutes. Visualization with DAB was achieved by adding ANS (0,3% final concentration) and hydrogen peroxide (0,0015% final concentration) for 15 minutes.
